# What exactly is Universal Grammar, and has anyone seen it?

**DOI:** 10.3389/fpsyg.2015.00852

**Published:** 2015-06-23

**Authors:** Ewa Dąbrowska

**Affiliations:** Department of Humanities, Northumbria University, Newcastle upon Tyne, UK

**Keywords:** Universal Grammar, language universals, poverty of the stimulus, convergence, individual differences, language acquisition, construction grammar, linguistic nativism

## Abstract

Universal Grammar (UG) is a suspect concept. There is little agreement on what exactly is in it; and the empirical evidence for it is very weak. This paper critically examines a variety of arguments that have been put forward as evidence for UG, focussing on the three most powerful ones: universality (all human languages share a number of properties), convergence (all language learners converge on the same grammar in spite of the fact that they are exposed to different input), and poverty of the stimulus (children know things about language which they could not have learned from the input available to them). I argue that these arguments are based on premises which are either false or unsubstantiated. Languages differ from each other in profound ways, and there are very few true universals, so the fundamental crosslinguistic fact that needs explaining is diversity, not universality. A number of recent studies have demonstrated the existence of considerable differences in adult native speakers’ knowledge of the grammar of their language, including aspects of inflectional morphology, passives, quantifiers, and a variety of more complex constructions, so learners do not in fact converge on the same grammar. Finally, the poverty of the stimulus argument presupposes that children acquire linguistic representations of the kind postulated by generative grammarians; constructionist grammars such as those proposed by Tomasello, Goldberg and others can be learned from the input. We are the only species that has language, so there must be something unique about humans that makes language learning possible. The extent of crosslinguistic diversity and the considerable individual differences in the rate, style and outcome of acquisition suggest that it is more promising to think in terms of a language-making capacity, i.e., a set of domain-general abilities, rather than an innate body of knowledge about the structural properties of the target system.

## Introduction

The Universal Grammar (UG) hypothesis—the idea that human languages, as superficially diverse as they are, share some fundamental similarities, and that these are attributable to innate principles unique to language: that deep down, there is only one human language (Chomsky, 2000a, p. 7)—has generated an enormous amount of interest in linguistics, psychology, philosophy, and other social and cognitive sciences. The predominant approach in linguistics for almost 50 years (Smith, 1999, p. 105: described it as “unassailable”), it is now coming under increasing criticism from a variety of sources. In this paper, I provide a critical assessment of the UG approach. I argue that there is little agreement on what UG actually is; that the arguments for its existence are either irrelevant, circular, or based on false premises; and that there are fundamental problems with the way its proponents address the key questions of linguistic theory.

## What Exactly is UG?

Universal Grammar is usually defined as the “system of categories, mechanisms and constraints shared by all human languages and considered to be innate” (O’Grady et al., 1996, p. 734; cf. also Chomsky, 1986, p. 3, 2007, p. 1; Pesetsky, 1999, p. 476). These are generally thought to include formal universals (e.g., principles, i.e., general statements which specify the constraints on the grammars of human languages, and parameters, which specify the options for grammatical variation between languages) as well as substantive universals (e.g., lexical categories and features). There is very little agreement, however, on what these actually are.

Chomsky (1986) sees UG as “an intricate and highly constrained structure” (p. 148) consisting of “various subsystems of principles” (p. 146). These include “X-bar theory, binding theory, Case theory, theta theory, bounding theory … and so forth – each containing certain principles with a limited degree of parametric variation. In addition there are certain overriding principles such as the projection principle, FI (full interpretation), and the principles of licensing… [UG also contains] certain concepts, such as the concept of domain … and the related notions of c-command and government” (p. 102). However, every major development in the theory since then was accompanied by very substantial revisions to the list of proposed universals. Thus the list of UG principles is quite different when we move to the Barriers period, and radically different in Minimalism (see below).

With respect to parameters, very few scholars have even attempted to give a reasonably comprehensive inventory of what these are. Two rare exceptions are Baker (2001), who discusses 10 parameters, and Fodor and Sakas (2004), who list 13. In both cases, the authors stress that the list is far from complete; but it is interesting to note that only three parameters occur on both lists (Tomasello, 2005; see also Haspelmath, 2007). There is no agreement even on approximately how many parameters there are: thus Pinker (1994, p. 112) claims that there are “only a few”; Fodor (2003, p. 734) suggests that there are “perhaps 20”; according to Roberts and Holmberg (2005, p. 541), the correct figure is probably “in the region of 50–100.” However, if, following Kayne (2005), we assume that there is a parameter associated with every functional element, the number of parameters must be considerably larger than this. Cinque and Rizzi (2008), citing Heine and Kuteva’s (2002) work on grammaticalization targets, estimate that there are about 400 functional categories. According to Shlonsky (2010, p. 424), even this may be a low estimate. Shlonsky (2010) also suggests that “[e]very feature is endowed with its own switchboard, consisting of half a dozen or so binary options” (p. 425), which implies that there are thousands of parameters.

Things are no better when we consider substantive universals. While most generative linguists agree that the inventory of lexical categories includes N, V, and A, there is little agreement on what the functional categories are (see Newmeyer, 2008; Corbett, 2010; Pullum and Tiede, 2010; Boeckx, 2011). Newmeyer (2008) surveys some of the relevant literature and concludes:

“There is no way to answer this question that would satisfy more than a small number of generativists. It seems fair to say that categories are proposed for a particular language when they appear to be needed for that language, with little thought as to their applicability to the grammar of other languages. My guess is that well over two hundred have been put forward in current work in the principles-and-parameters tradition.” (p. 51)

The situation, Newmeyer (2008) observes, is even less clear when it comes to features:

“Even more than for categories, features tend to be proposed ad hoc in the analysis of a particular language when some formal device is needed to distinguish one structure (or operation on a particular structure) from another. As a result, supplying even a provisional list of what the set of universal distinctive syntactic features might be seems quite hopeless.” (p. 53)

Thus, some linguists see UG as a very elaborate structure, consisting of a large number of principles, parameters, and categories. At the other extreme, we have the strong minimalist thesis, according to which UG may comprise just the structure-building operation Merge (cf. Chomsky, 2004, 2012; Berwick et al., 2011). It seems that the only point of agreement amongst proponents of UG is that it exists; they do not agree on what it actually contains. What evidence, then, is there for the existence of specifically linguistic innate knowledge? I turn to this question in the next section.

## Arguments for UG

Over the years, a number of arguments have been put forward in support of the UG hypothesis. These include the following:

(1) Language Universals: (All) human languages share certain properties.
(2) Convergence: Children are exposed to different input yet converge on the same grammar.
(3) Poverty of the Stimulus: Children acquire knowledge for which there is no evidence in the input.
(4) No Negative Evidence: Children know which structures are ungrammatical and do not acquire overgeneral grammars in spite of the fact that they are not exposed to negative evidence.
(5) Species Specificity: We are the only species that has language.
(6) Ease and Speed of Child Language Acquisition: Children learn language quickly and effortlessly, on minimal exposure.
(7) Uniformity: All children acquiring language go through the same stages in the same order.
(8) Maturational Effects: Language acquisition is very sensitive to maturational factors and relatively insensitive to environmental factors.
(9) Dissociations between Language and Cognition: Some clinical populations have (relatively) normal language and impaired cognition; some have impaired cognition and (relatively) normal language.
(10) Neurological Separation: Different brain circuits are responsible for representing/processing linguistic and non-linguistic information.

Arguments 1–4 are generally regarded as the most powerful ones; 5–10 are subsidiary in the sense they only provide support for the idea of innateness of language general, rather than the innateness of a specific aspect of linguistic organization, and they are also open to other interpretations. I begin by evaluating the subsidiary arguments, and then move on to the more powerful ones.

### Species Specificity

“To say that language is not innate is to say that there is no difference between my granddaughter, a rock and a rabbit. In other words, if you take a rock, a rabbit and my granddaughter and put them in a community where people are talking English, they’ll all learn English. If people believe that, then they believe that language is not innate. If they believe that there is a difference between my granddaughter, a rabbit, and a rock, then they believe that language is innate.” (Chomsky, 2000b, p. 50)

Clearly, there is something unique about human biological make-up that makes it possible for humans, and only humans, to acquire language. However, *nobody* disputes this, so in the passage quoted above Chomsky is fighting a straw man. The crucial question is whether the relevant knowledge or abilities are language-specific or whether they can be attributed to more general cognitive processes—and this is far from clear.

There are a number of other characteristics which appear to be specific to our species. These include collaboration, cultural learning, the use of complex tools, and—surprisingly—the use of pointing and others means of drawing attention to particular features of the immediate environment, such as holding objects up for others to see.^1^ This suggests there may be a more fundamental difference between humans and the rest of the animal kingdom. As Tomasello et al. (2005) put it, “saying that only humans have language is like saying that only humans build skyscrapers, when the fact is that only humans (among primates) build freestanding shelters at all” (p. 690). Tomasello et al. (2005) argue that language is a consequence of the basic human ability to recognize others’ communicative intentions and to engage in joint attention, which also underlies other cultural achievements.

The ability to read and share intentions, including communicative intentions—i.e., theory of mind in the broad sense—is important for language for two reasons. First, it enables the language learner to understand what language is *for*: an animal that did not understand that other individuals have beliefs and intentions different from its own would have little use for language. Secondly, it provides the learner with a vital tool for learning language. In order to learn a language, one must acquire a set of form-meaning conventions; and to acquire these, learners must be able to guess at least some of the meanings conveyed by the utterances they hear.

The human ability to read and share intentions may not explain subjacency effects—the existence of other differences between humans and other species does not entail lack of UG, just as species specificity does not entail its existence. The point is that arguments for the innateness of language in a general sense (what Scholz and Pullum, 2002 call “general nativism”) do not constitute arguments for the innateness of UG (“linguistic nativism”) if UG is taken to be a specific body of linguistic knowledge. In other words, the fact that we are the only species that has language does not entail that we have innate knowledge of subjacency.

### Ease and Speed of Child Language Acquisition

It has been often suggested that children acquire grammatical systems of enormous complexity rapidly and effortlessly on the basis of very little evidence, and by “mere exposure,” that is to say, without explicit teaching (see, for example, Chomsky, 1962, p. 529, 1976, p. 286, 1999; Guasti, 2002, p. 3). In fact, they get vast amounts of language experience. If we assume that language acquisition begins at age 1 and ends at age 5 and that children are exposed to language for 8 h a day, they get 11680 h of exposure (4 × 365 × 8 = 11680). At 3600 input words per hour (the average number of words heard by the children in the Manchester corpus),^2^ this amounts to over 42 million words over 4 years.

Note that this is a rather conservative estimate: we know that language development begins before age 1 (Jusczyk, 1997; Karmiloff and Karmiloff-Smith, 2001) and continues throughout childhood and adolescence (Nippold, 1998; Berman, 2004, 2007; Nippold et al., 2005; Kaplan and Berman, 2015); moreover, children are exposed to language—through utterances directed to them, utterances directed to other people present, radio and television, and later school, reading and the internet almost every waking hour of their lives.

Furthermore, we know that “mere exposure” is not enough—as demonstrated by studies of hearing children of deaf parents (Todd and Aitchison, 1980; Sachs et al., 1981; see also Dąbrowska, 2012, for some observations on the effects of the quality of the input). Consider, for example, Jim—one of children studied by Sachs et al. (1981). In early childhood, Jim had very little contact with hearing adults but watched television quite frequently, and occasionally played with hearing children. His parents used sign language when addressing each other, but did not sign to the children. At age 3;9 (3 years and 9 months)—the beginning of the study—Jim had very poor comprehension of spoken language, and severe articulation problems. His utterances were very short, with an MLU (mean length of utterance) of 2.9—typical for a child aged about 2;9. He had low use of grammatical morphemes, producing them in only 37% of obligatory contexts, while MLU-matched controls supplied them 64–81% of the time; and many of his utterances had clearly deviant syntax (My mommy my house ǝ play ball; House ǝ chimney my house ǝ my chimney). And, interestingly, although he was exposed to ASL at home, he did not sign. Jim’s spoken language improved rapidly once he began interacting with adults on a one-on-one basis, and by age 6;11, he performed above age level on most measures—showing that he was not language impaired. Thus, although he was exposed to both spoken English (through television and occasional interaction with other children) and to ASL (though observing his parents), Jim did not acquire either language until he was given an opportunity to interact with competent users.

### Uniformity

Some researchers (e.g., Stromswold, 2000; Guasti, 2002) have suggested that children acquire language in a very similar manner, going through the same stages at approximately the same ages, in spite of the fact that they are exposed to different input. Stromswold (2000), for instance, observes that

“Within a given language, the course of language acquisition is remarkably uniform…. Most children say their first referential words at 9 to 15 months… and for the next 6-8 months, children typically acquire single words fairly slowly until they have acquired approximately 50 words…. Once children have acquired 50 words, their vocabularies often increase rapidly…. At around 18 to 24 months, children learning morphologically impoverished languages such as English begin combining words to form two-word utterances…. Children acquiring such morphologically impoverished languages gradually begin to use sentences longer than two words; but for several months their speech often lacks phonetically unstressed functional category morphemes such as determiners, auxiliary verbs, and verbal and nominal inflectional endings …. Gradually, omissions become rarer until children are between three and four years old, at which point the vast majority of English-speaking children’s utterances are completely grammatical.” (p. 910)

This uniformity, Stromswold argues, indicates that the course of language acquisition is strongly predetermined by an innate program.

There are several points to be made in connection with this argument. First, many of the similarities that Stromswold mentions are not very remarkable: we do not need UG to explain why children typically (though by no means always) produce single word utterances before they produce word combinations, or why frequent content words are acquired earlier than function words. Secondly, the age ranges she gives (e.g., 9–15 months for first referential words) are quite wide: 6 months is a very long time for an infant. Thirdly, the passage describes *typical* development, as evidenced by qualifiers like “most children,” “typically,” “often”—so the observations are not true of all children. Finally, by using qualifiers like “within a given language” and limiting her observations to “children acquiring morphologically impoverished languages” Stromswold implicitly concedes the existence of crosslinguistic differences. These are quite substantial: children acquiring different languages have to rely on different cues, and this results in different courses of development (Bavin, 1995; Jusczyk, 1997; Lieven, 1997); and they often acquire “the same” constructions at very different ages. For example, the passive is acquired quite late by English speaking children—typically (though by no means always—see below) by age 4 or 5, and even later—by about 8—by Hebrew-speaking children (Berman, 1985). However, children learning languages in which the passive is more frequent and/or simpler master this construction much earlier—by about 2;8 in Sesotho (Demuth, 1989) and as early as 2;0 in Inuktitut (Allen and Crago, 1996).

Even within the same language, contrary to Stromswold’s claims, there are vast individual differences both in the rate and course of language development (Bates et al., 1988; Richards, 1990; Shore, 1995; Goldfield and Snow, 1997; Peters, 1997; Huttenlocher, 1998). Such differences are most obvious, and easiest to quantify, in lexical development. The comprehension vocabularies of normally developing children of the same age can differ tenfold or more (Benedict, 1979; Goldfield and Reznick, 1990; Bates et al., 1995). There are also very large differences in the relationship between a child’s expressive and receptive vocabulary early in development: some children are able to understand over 200 words before they start producing words themselves, while others are able to produce almost all the words they know (Bates et al., 1995). Children also differ with regard to the kinds of words they learn in the initial stages of lexical development. “Referential” children initially focus primarily on object labels (i.e., concrete nouns), while “expressive” children have more varied vocabularies with more adjectives and verbs and some formulaic phrases such as *thank you*, *not now*, *you’re kidding*, *don’t know* (Nelson, 1973, 1981). Last but not least, there are differences in the pattern of growth. Many children do go through the “vocabulary spurt” that Stromswold alludes to some time between 14 and 22 months, but about a quarter do not, showing a more gradual growth pattern with no spurt (Goldfield and Reznick, 1990).

Grammatical development is also far from uniform. While some children begin to combine words as early as 14 months, others do not do so until after their second birthday (Bates et al., 1995), with correspondingly large differences in MLU later in development—from 1.2 to 5.0 at 30 months (Wells, 1985). Some children learn to inflect words before they combine them into larger structures, while others begin to combine words before they are able to use morphological rules productively (Smoczyńska, 1985, p. 618; Thal et al., 1996). Some children are very cautious learners who avoid producing forms they are not sure about, while others are happy to generalize on the basis of very little evidence. This results in large differences in error rates (Maratsos, 2000). Considerable individual differences have also been found in almost every area of grammatical development where researchers have looked for them, including word order (Clark, 1985), case marking (Dąbrowska and Szczerbiński, 2006), the order of emergence of grammatical morphemes (Brown, 1973), auxiliary verbs (Wells, 1979; Richards, 1990; Jones, 1996), questions (Gullo, 1981; Kuczaj and Maratsos, 1983; de Villiers and de Villiers, 1985), passives (Horgan, 1978; Fox and Grodzinsky, 1998), and multiclause sentences (Huttenlocher et al., 2002).

Children also differ in their learning “styles” (Peters, 1977; Nelson, 1981; Peters and Menn, 1993). “Analytic” (or “referential”) children begin with single words, which they articulate reasonably clearly and consistently. “Holistic” (or “expressive”) children, on the other hand, begin with larger units which have characteristic stress and intonation patterns, but which are often pronounced indistinctly, and sometimes consist partly or even entirely of filler syllables such as [dadada]. Peters (1977) argues that holistic children attempt to approximate the overall shape of the target utterance while analytic children concentrate on extracting and producing single words. These different starting points determine how the child “breaks into” grammar, and therefore have a substantial effect on the course of language development. Analytic children must learn how to combine words to form more complex units. They start by putting together content words, producing telegraphic utterances such as *there doggie* or *doggie eating*. Later in development they discover that different classes of content words require specific function words and inflections (nouns take determiners, verbs take auxiliaries, and tense inflections, etc.), and gradually learn to supply these. Holistic children, in contrast, must segment their rote-learned phrases and determine how each part contributes to the meaning of the whole. Unlike analytic children, they sometimes produce grammatical morphemes very early in acquisition, embedded in larger unanalysed or only partially analyzed units; or they may use filler syllables as place-holders for grammatical morphemes. As their systems develop, the fillers gradually acquire more phonetic substance and an adult-like distribution, and eventually evolve into function words of the target language (Peters and Menn, 1993; Peters, 2001). Thus, while both groups of children eventually acquire similar grammars, they get there by following different routes.^3^

### Maturational Effects

Language acquisition is sometimes claimed to be “highly sensitive to maturational factors” and “surprisingly insensitive to environmental factors” (Fodor, 1983, p. 100; see also Gleitman, 1981; Crain and Lillo-Martin, 1999; Stromswold, 2000), which, these researchers suggest, indicates that the language faculty develops, or matures, according to a biologically determined timetable.

The claim that language acquisition is insensitive to environmental factors is simply incorrect, as demonstrated by the vast amount of research showing that both the amount and quality of input have a considerable effect on acquisition—particularly for vocabulary, but also for grammar (e.g., Huttenlocher, 1998; Huttenlocher et al., 2002; Ginsborg, 2006; Hoff, 2006). There is no doubt that maturation also plays a very important role—but this could be due to the development of the cognitive prerequisites for language (Slobin, 1973, 1985; Tomasello, 2003) rather than the maturation of the language faculty. Likewise, while it is possible that critical/sensitive period effects are due to UG becoming inaccessible at some point in development, they could also arise as a result of older learners’ greater reliance on declarative memory (Ullman, 2006), developmental changes in working memory capacity (Newport, 1990), or entrenchment of earlier learning (Elman et al., 1996; MacWhinney, 2008). Thus, again, the existence of maturational effects does not entail the existence of an innate UG: they are, at best, an argument for general innateness, not linguistic innateness.

### Dissociations between Language and Cognition

A number of researchers have pointed out that some individuals (e.g., aphasics and children with Specific Language Impairment) show severe language impairment and relatively normal cognition, while others (e.g., individuals with Williams syndrome (WS), or Christopher, the “linguistic savant” studied by Smith and Tsimpli, 1995) show the opposite pattern: impaired cognition but good language skills. The existence of such a double dissociation suggests that language is not part of “general cognition”—in other words, that it depends at least in part on a specialized linguistic “module.”

The existence of double dissociations in adults is not particularly informative with regard to the innateness issue, however, since modularization can be a result of development (Paterson et al., 1999; Thomas and Karmiloff-Smith, 2002); hence, the fact that language is relatively separable in adults does not entail innate linguistic knowledge. On the other hand, the developmental double dissociation between specific language impairment (SLI) and WS, is, on the face of it, much more convincing. There are, however, several reasons to be cautious in drawing conclusions from the observed dissociations.

First, there is growing evidence suggesting that WS language is impaired, particularly early in development (Karmiloff-Smith et al., 1997; Brock, 2007; Karmiloff-Smith, 2008). Children with WS begin talking much later than typically developing children, and their language develops along a different trajectory. Adolescents and adults with WS show deficits in all areas of language: syntax (Grant et al., 2002), morphology (Thomas et al., 2001), phonology (Grant et al., 1997), lexical knowledge (Temple et al., 2002), and pragmatics (Laws and Bishop, 2004). Secondly, many, perhaps all, SLI children have various non-linguistic impairments (Leonard, 1998; Tallal, 2003; Lum et al., 2010)—making the term *Specific* Language Impairment something of a misnomer. Thus the dissociation is, at best, partial: older WS children and adolescents have relatively good language in spite of a severe cognitive deficit; SLI is a primarily linguistic impairment.

More importantly, it is debatable whether we are really dealing with a double dissociation in this case. Early reports of the double dissociation between language and cognition in Williams and SLI were based on indirect comparisons between the two populations. For instance, Pinker (1999) discusses a study conducted by Bellugi et al. (1994), which compared WS and Down’s syndrome adolescents and found that the former have much better language skills, and van der Lely’s work on somewhat younger children with SLI (van der Lely, 1997; van der Lely and Ullman, 2001), which found that SLI children perform less well than typically developing children. However, a study which compared the two populations directly (Stojanovik et al., 2004) suggests rather different conclusions. Stojanovik et al. (2004) gave SLI and WS children a battery of verbal and non-verbal tests. As expected, the SLI children performed much better than the WS children on all non-verbal measures. However, there were no differences between the two groups on the language tests—in fact, the SLI children performed slightly better on some measures, although the differences were not statistically significant. Clearly, one cannot argue that language is selectively impaired in SLI and intact in WS if we find that the two populations’ performance on the same linguistic tests is indistinguishable.

To summarize: There is evidence of a partial dissociation in SLI children, who have normal IQ and below-normal language—and, as pointed out earlier, a variety of non-linguistic impairments which may the underlying cause of their linguistic deficit. There is, however, no evidence for a dissociation in Williams syndrome: WS children’s performance on language tests is typically appropriate for their mental age, and well below their chronological age.

### Neurological Separation

The fact that certain parts of the brain—specifically, the perisylvian region including Broca’s area, Wernicke’s area and the angular gyrus—appear to be specialized for language processing has led some researchers (e.g., Pinker, 1995; Stromswold et al., 1996; Stromswold, 2000, p. 925; Musso et al., 2003) to speculate that they may constitute the neural substrate for UG. Intriguing though such proposals are, they face a number of problems. First, the language functions are not strongly localized: many other areas outside the classical “language areas” are active during language processing; and, conversely, the language areas may also be activated during non-linguistic processing (Stowe et al., 2005; Anderson, 2010; see, however, Fedorenko et al., 2011). More importantly, studies of neural development clearly show that the details of local connectivity in the language areas (as well as other areas of the brain) are not genetically specified but emerge as a result of activity and their position in the larger functional networks in the brain (Elman et al., 1996; Müller, 2009; Anderson et al., 2011; Kolb and Gibb, 2011). Because of this, human brains show a high amount of plasticity, and other areas of the brain can take over if the regions normally responsible for language are damaged. In fact, if the damage occurs before the onset of language, most children develop normal conversational skills (Bates et al., 1997; Aram, 1998; Bates, 1999; Trauner et al., 2013), although language development is often delayed (Vicari et al., 2000), and careful investigations do sometimes reveal residual deficits in more complex aspects of language use (Stiles et al., 2005; Reilly et al., 2013). Lesions sustained in middle and late childhood typically leave more lasting deficits, although these are relatively minor (van Hout, 1991; Bishop, 1993; Martins and Ferro, 1993). In adults, the prospects are less good, but even adults typically show some recovery (Holland et al., 1996), due partly to regeneration of the damaged areas and partly to shift to other areas of the brain, including the right hemisphere (Karbe et al., 1998; Anglade et al., 2014). Thus, while the neurological evidence does suggest that certain areas of the brain are particularly well-suited for language processing, there is no evidence that these regions actually contain a genetically specified preprint blueprint for grammar.

### Language Universals

Generative linguists have tended to downplay the differences between languages and emphasize their similarities. In Chomsky’s (2000a) words,

“… in their essential properties and even down to fine detail, languages are cast to the same mold. The Martian scientist might reasonably conclude that there is a single human language, with differences only at the margins.” (p. 7)

Elsewhere (Chomsky, 2004, p. 149) he describes human languages as “essentially identical.” Stromswold (1999) expresses virtually the same view:

“In fact, linguists have discovered that, although some languages seem, superficially, to be radically different from other languages …, in essential ways all human languages are remarkably similar to one another.” (p. 357)

This view, however, is not shared by most typologists (cf. Croft, 2001; Haspelmath, 2007; Evans and Levinson, 2009). Evans and Levinson (2009), for example, give counterexamples to virtually all proposed universals, including major lexical categories, major phrasal categories, phrase structure rules, grammaticalized means of distinguishing between subjects and objects, use of verb affixes to signal tense and aspect, auxiliaries, anaphora, and WH movement, and conclude that

“….languages differ so fundamentally from one another at every level of description (sound, grammar, lexicon, meaning) that it is very hard to find any single structural property they share. The claims of Universal Grammar … are either empirically false, unfalsifiable or misleading in that they refer to tendencies rather than strict universals.” (p. 429)

Clearly, there is a fundamental disagreement between generative linguists like Chomsky and functionalists like Evans and Levinson (2009). Thus, it is misleading to state that “linguists have discovered that … in essential ways all human languages are remarkably similar to one another”; it would have been more accurate to prefix such claims with a qualifier such as “some linguists think that….”

One reason for the disagreement is that generative and functional linguists have a very different view of language universals. For the functionalists, universals are inductive generalizations about observable features of language, discovered by studying a large number of unrelated languages—what some people call descriptive, or “surface” universals. The generativists’ universals, on the other hand, are cognitive or “deep” universals, which are highly abstract and cannot be derived inductively from observation of surface features. As Smolensky and Dupoux (2009) argue in their commentary on Evans and Levinson’s paper,

“Counterexamples to des-universals are not counterexamples to cog-universals … a hypothesised cog-universal can only be falsified by engaging the full apparatus of the formal theory.” (p. 468)

This is all very well—but how exactly do we “engage the full apparatus of the formal theory”? The problem with deep universals is that in order to evaluate them, one has to make a number of subsidiary (and often controversial) assumptions which in turn depend on further assumptions—so the chain of reasoning is very long indeed (cf. Hulst, 2008; Newmeyer, 2008). This raises obvious problems of falsifiability. Given that most deep universals are parameterized, that they may be parameterized “invisibly,” and that some languages have been argued to be exempt from some universals (cf. Newmeyer, 2008), it is not clear what would count as counterevidence for a proposed universal.

The issue is particularly problematic for substantive universals. The predominant view of substantive universals (lexical categories, features, etc.,) is that they are part of UG, but need not be used by all languages: in other words, UG makes available a list of categories, and languages “select” from this list. But as Evans and Levinson (2009) point out,

“… the claim that property X is a substantive universal cannot be falsified by finding a language without it, because the property is not required in all of them. Conversely, suppose we find a new language with property Y, hitherto unexpected: we can simply add it to the inventory of substantive universals…. without limits on the toolkit, UG is unfalsifiable.” (p. 436)

Apart from issues of falsifiability, the fact that deep universals are theory internal has another consequence, nicely spelled out by Tomasello (1995):

“Many of the Generative Grammar structures that are found in English can be found in other languages—if it is generative grammarians who are doing the looking. But these structures may not be found by linguists of other theoretical persuasions because these structures are defined differently, or not recognised at all, in other linguistic theories.” (p. 138)

In other words, deep universals may exist—but they cannot be treated as evidence for the theory, because they are assumed by the theory.

Returning to the more mundane, observable surface universals: although absolute universals are very hard to find, there is no question that there are some very strong universal tendencies, and these call for an explanation. Many surface universals have plausible functional explanations (Comrie, 1983; Hawkins, 2004; Haspelmath, 2008). It is also possible that they derive from a shared protolanguage or that they are in some sense “innate,” i.e., that they are part of the initial state of the language faculty—although existing theories of UG do not fare very well in explaining surface universals (Newmeyer, 2008).

Generative linguists’ focus on universals has shifted attention from what may be the most remarkable property of human languages—their diversity. Whatever one’s beliefs about UG and the innateness hypothesis, it is undeniable that some aspects of our knowledge—the lexicon, morphological classes, various idiosyncratic constructions, i.e., what generative linguists sometimes refer to as the “periphery”—must be learned, precisely because they are idiosyncratic and specific to particular languages. These aspects of our linguistic knowledge are no less complex (in fact, in some cases considerably more complex) than the phenomena covered by “core” grammar, and mastering them requires powerful learning mechanisms. It is possible, then, that the cognitive mechanisms necessary to learn about the periphery may suffice to learn core grammar as well (Menn, 1996; Culicover, 1999; Dąbrowska, 2000a).

### Convergence

“… it is clear that the language each person acquires is a rich complex construction hopelessly underdetermined by the fragmentary evidence available [to the learner]. Nevertheless individuals in a speech community have developed essentially the same language. This fact can be explained only on the assumption that these individuals employ highly restrictive principles that guide the construction of the grammar.” (Chomsky, 1975, p. 11)

“The set of utterances to which any child acquiring a language is exposed is equally compatible with many distinct descriptions. And yet children converge to a remarkable degree on a common grammar, with agreement on indefinitely many sentences that are novel. Mainly for this reason, Chomsky proposed that the child brings prior biases to the task.” (Lidz and Williams, 2009, p. 177)

“The explanation that is offered must also be responsive to other facts about the acquisition process; in particular, the fact that every child rapidly converges on a grammatical system that is equivalent to everyone else’s, despite a considerable latitude in linguistic experience – indeed, without any relevant experience in some cases. Innate formal principles of language acquisition are clearly needed to explain these basic facts.” (Crain et al., 2009, p. 124)

As illustrated by these passages, the (presumed) fact that language learners converge on the same grammar despite having been exposed to different input is often regarded as a powerful argument for an innate UG. It is interesting to note that all three authors quoted above simply assume that learners acquire essentially the same grammar: the convergence claim is taken as self-evident, and is not supported with any evidence. However, a number of recent studies which have investigated the question empirically found considerable individual differences in how much adult native speakers know about the grammar of their language, including inflectional morphology (Indefrey and Goebel, 1993; Dąbrowska, 2008), a variety of complex syntactic structures involving subordination (Dąbrowska, 1997, 2013; Chipere, 2001, 2003), and even simpler structures such as passives and quantified noun phrases (Dąbrowska and Street, 2006; Street, 2010; Street and Dąbrowska, 2010, 2014; for recent reviews, see Dąbrowska, 2012, 2015).

For example, Street and Dąbrowska (2010) tested adult native English speakers’ comprehension of simple sentences with universal quantifiers such as (1–2) and unbiased passives (3); the corresponding actives (4) were a control condition.

(1) Every toothbrush is in a mug.
(2) Every mug has a toothbrush in it.
(3) The girl was hugged by the boy.
(4) The girl hugged the boy.

Participants listened to each test sentence and were asked to select the matching picture from an array of two. For the quantifier sentences the pictures depicted objects and containers in partial one-to-one correspondence (e.g., three mugs, each with a toothbrush in it plus an extra toothbrush; three mugs, each with a toothbrush in it plus an extra mug). For actives and passives, the pictures depicted a transitive event (e.g., a girl hugging a boy and a boy hugging a girl).

Experiment 1 tested two groups, a high academic attainment (HAA) group, i.e., postgraduate students, and a low academic attainment (LAA) group, who worked as shelf-stackers, packers, assemblers, or clerical workers and who had no more than 11 years of formal education. The HAA participants consistently chose the target picture in all four conditions. The LAA participants were at ceiling on actives, 88% correct on passives, 78% on simple locatives with quantifiers, and 43% correct (i.e., at chance) on possessive locatives with quantifiers. The means for the LAA group mask vast differences between participants: individual scores in this group ranged from 0 to 100% for the quantifier sentences and from 33 to 100% for passives.

Street and Dąbrowska argue that the experiment reveals differences in linguistic knowledge (competence), not performance, pointing out that the picture selection task has minimal cognitive demands (and can be used with children as young as 2 to test simpler structures); moreover, all participants, including the LAA group, were at ceiling on active sentences, showing that they had understood the task, were cooperative, etc. (For further discussion of this issue, see Dąbrowska, 2012.)

Experiment 2 was a training study. LAA participants who had difficulty with all three of the experimental constructions (i.e., those who scored no more than 4 out of 6 correct on each construction in the pre-test) were randomly assigned to either a passive training group or a quantifier training group. The training involved an explicit explanation of the target construction followed by practice with feedback. Subsequently, participants were given a series of post-tests: immediately after training, a week later, and 12 weeks after training. The results revealed that performance improved dramatically after training, but only on the construction trained, and that the effects of training were long-lasting—that is to say, the participants performed virtually at ceiling even on the last post-test. This indicates that the participants were not language impaired, and that their poor performance on the pre-test is attributable to lack of knowledge rather than failure to understand the instructions or to cooperate with the experimenter.

The existence of individual differences in linguistic attainment is not, of course, incompatible with the existence of innate predispositions and biases. In fact, we know that differences in verbal ability are heritable (Stromswold, 2001; Misyak and Christiansen, 2011), although it is clear that environmental factors also play an important role (see Dąbrowska, 2012). However, the Street and Dąbrowska experiments as well as other studies mentioned earlier in this section suggest that the convergence argument is based on a false premise. Native speakers do not converge on the same grammar: there are, in fact, considerable differences in how much speakers know about some of the basic constructions of their native language.

### Poverty of the Stimulus and Negative Evidence

The most famous, and most powerful, argument for UG is the poverty of the stimulus argument: the claim that children have linguistic knowledge which could not have been acquired from the input which is available to them:

“…every child comes to know facts about the language for which there is no decisive evidence from the environment. In some cases, there appears to be no evidence at all.” (Crain, 1991)

“People attain knowledge of the structure of their language for which no evidence is available in the data to which they are exposed as children.” (Hornstein and Lightfoot, 1981, p. 9)

“Universal Grammar provides representations that support deductions about sentences that fall outside of experience…. These abstract representations drive the language learner’s capacity to project beyond experience in highly specific ways.” (Lidz and Gagliardi, 2015)

The textbook example of the poverty of the stimulus is the acquisition of the auxiliary placement rule in English Y/N questions (see, for example, Chomsky, 1972, 2012; Crain, 1991; Lasnik and Uriagereka, 2002; Berwick et al., 2011). On hearing pairs of sentences such as (5a) and (5b) a child could infer the following rule for deriving questions:

Hypothesis A: Move the auxiliary to the beginning of the sentence.

However, such a rule would incorrectly derive (6b), although the only grammatical counterpart of (6a) is (6c).

5a The boy will win.
5b Will the boy win?
6a The boy who can swim will win.
6b *Can the boy who swim will win?
6c Will the boy who can swim win?

In order to acquire English, the child must postulate a more complex, structure dependent rule:

Hypothesis B: Move the first auxiliary after the subject to the beginning of the sentence.

Crucially, the argument goes, children never produce questions such as (6b), and they know that such sentences are ungrammatical; furthermore, it has been claimed that they know this without ever being exposed to sentences like (6c) (see, for example, Piattelli-Palmarini, 1980, p. 40, pp. 114–115; Crain, 1991).

A related issue, sometimes conflated with poverty of the stimulus, is lack of negative evidence. Language learners must generalize beyond the data that they are exposed to, but they must not generalize too much. A learner who assumed an overly general grammar would need negative evidence—evidence that some of the sentences that his or her grammar generates are ungrammatical—to bring the grammar in line with that of the speech community. Since such evidence is not generally available, learners’ generalizations must be constrained by UG (Baker, 1979; Marcus, 1993).

Let us begin with the negative evidence problem. Several observations are in order. First, while parents do not reliably correct their children’s errors, children do get a considerable amount of *indirect* negative evidence in the form of requests for clarification and adult reformulations of their erroneous utterances. Moreover, a number of studies have demonstrated that children understand that requests for clarification and recasts are negative evidence, and respond appropriately, and that corrective feedback results in improvement in the grammaticality of child speech (Demetras et al., 1986; Saxton et al., 1998; Saxton, 2000; Chouinard and Clark, 2003). Negative evidence can also be inferred from absence of positive evidence: a probabilistic learner can distinguish between accidental non-occurrence and a non-occurrence that is statistically significant, and infer that the latter is ungrammatical (Robenalt and Goldberg, in press; Scholz and Pullum, 2002, 2006; Stefanowitsch, 2008).

Secondly, as Cowie (2008) points out, the acquisition of grammar is not the only area where we have to acquire knowledge about what is not permissible without the benefit of negative evidence. We face exactly the same problem in lexical learning and learning from experience generally: few people have been explicitly told that custard is not ice-cream, and yet somehow they manage to learn this. Related to this, children do make overgeneralization errors—including morphological overgeneralizations like *bringed* and *gooder* and overgeneralizations of various sentence level constructions (e.g., *I said her no*, *She giggled me*), and they do recover from them (cf. Bowerman, 1988). Thus, the question isn’t “What sort of innate constraints must we assume to prevent children from overgeneralizing?” but rather “How do children recover from overgeneralization errors?”—and there is a considerable amount of research addressing this very issue (see, for example, Brooks and Tomasello, 1999; Brooks et al., 1999; Tomasello, 2003; Ambridge et al., 2008, 2009, 2011; Boyd and Goldberg, 2011).

Let us return to the poverty of the stimulus argument. The structure of the argument may be summarized as follows:

(1) Children know certain things about language.
(2) To learn them from the input, they would need access to data of a particular kind.
(3) The relevant data is not available in the input, or not frequent enough in the input to guarantee learning.
(4) Therefore, the knowledge must be innate.

As with any deductive argument, the truth of the conclusion (4) depends on the validity of the argument itself and the truth of the premises. Strikingly, most expositions of the poverty of the stimulus argument in the literature do not take the trouble to establish the truth of the premises: it is simply assumed. In a well-known critique of the POS argument, Pullum and Scholz (2002) analyze four linguistic phenomena (plurals inside compounds, anaphoric *one*, auxiliary sequences, auxiliary placement in Y/N questions) which are most often used to exemplify it, and show that the argument does not hold up: in all four cases, either the generalization that linguists assumed children acquired is incorrect or the relevant data is present in the input, or both. With respect to the auxiliary placement rule, for example, Pullum and Scholz (2002) estimate that by age 3, most children will have heard between 7500 and 22000 utterances that falsify the structure independent rule.

Lasnik and Uriagereka (2002) and others argue that Pullum and Scholz (2002) have missed the point: knowing that sentences like (6c) are grammatical does not entail that sentences like (6b) are not; and it does not tell the child how to actually form a question. They point out that “not even the fact that [6c] is grammatical proves that something with the effect of hypothesis B is correct (*and the only possibility* [my italics]), hence does not lead to adult knowledge of English” (Lasnik and Uriagereka, 2002; p. 148), and conclude that “children come equipped with a priori knowledge of language… because it is *unimaginable* [my italics] how they could otherwise acquire the complexities of adult language” (pp. 149–150).

Note that Lasnik and Uriagereka (2002) have moved beyond the original poverty of the stimulus argument. They are not arguing merely that a particular aspect of our linguistic knowledge must be innate because the relevant data is not available to learners (poverty of the stimulus); they are making a different argument, which Slobin (cited in Van Valin, 1994) refers to as the “argument from the poverty of the imagination”: “I can’t imagine how X could possibly be learned from the input; therefore, it must be innate.” Appeals to lack of imagination are not very convincing, however. One can easily construct analogous arguments to argue for the opposite claim: “I can’t imagine how X could have evolved (or how it could be encoded in the genes); therefore, it must be learned.” Moreover, other researchers may be more imaginative.

## The Construction Grammar Approach

Lasnik and Uriagereka (2002) conclude their paper with a challenge to non-nativist researchers to develop an account of how grammar could be learned from positive evidence. The challenge has been taken up by a number of constructionist researchers (Tomasello, 2003, 2006; Dąbrowska, 2004; Goldberg, 2006; for reviews, see Diessel, 2013; Matthews and Krajewski, 2015). Let us begin by examining how a constructionist might account for the acquisition of the auxiliary placement rule.

### Case Study: The Acquisition of Y/N Questions by Naomi

Consider the development of Y/N questions with the auxiliary *can* in one particular child, Naomi (see Dąbrowska, 2000b, 2004, 2010a, also discussed data for two other children from the CHILDES database).^4^ The first recorded questions with *can* appeared in Naomi’s speech at age 1;11.9 (1 year, 11 months and 9 days) and were correctly inverted:

1; 11.9 can I get down? [repeated 4x]
1; 11.9 can I get up?

Seven days later there are some further examples, but this time the subject is left out, although it is clear from the context that the subject is Naomi herself:

1; 11.16 can eat it ice cream?
1; 11.16 can lie down? [repeated 2x]

In total, there are 56 tokens of this “permission formula” in the corpus, 25 with explicit subjects.

The early questions with *can* are extremely stereotypical: the auxiliary is always placed at the beginning of the sentence (there are no “uninverted” questions), and although the first person pronoun is often left out, the agent of the action is invariably Naomi herself. There are other interesting restrictions on her usage during this period. For example, in Y/N interrogatives with *can*, if she explicitly refers to herself, she always uses the pronoun *I* (25 tokens)—never her name. In contrast, in other questions (e.g., the formulas *What’s Nomi do?*, *What’s Nomi doing?*, and *Where’s Nomi?*—45 tokens in total) she always refers to herself as *Nomi*. Furthermore, while she consistently inverts in first person questions with *can* and *could*, all the other Y/N questions with first person subjects are uninverted.

As the formula is analyzed, usage becomes more flexible. Two weeks after the original *can I…?* question, a variant appears with *could* instead of *can*:

1; 11.21 could do this?
2; 0.3 could I throw that?

Five weeks later, we get the first question with a subject other than *I*:

2; 0.28 can you draw eyes?

The transcripts up to this point contain 39 questions with *can*, including 10 with explicit subjects.

So we see a clear progression from an invariant formula (*Can I get down?*) through increasingly abstract formulaic frames (*Can I* + ACTION? ABILITY VERB + *I* + ACTION?) to a fairly general constructional schema in which none of the slots is tied to particular lexical items (ABILITY VERB + PERSON + ACTION?).

Questions with other auxiliaries follow different developmental paths. Not surprisingly, the first interrogatives with *will* were requests (*will you ACTION?*); this was later generalized to questions about future actions, and to other agents (*will* PERSON ACTION?). The earliest interrogatives with *do* were offers of a specific object (*do you want THING?*). This was later generalized to *do you ACTION?*; but for a long time, Naomi used “*do* support” almost exclusively with second person subjects.

**FIGURE 1:**
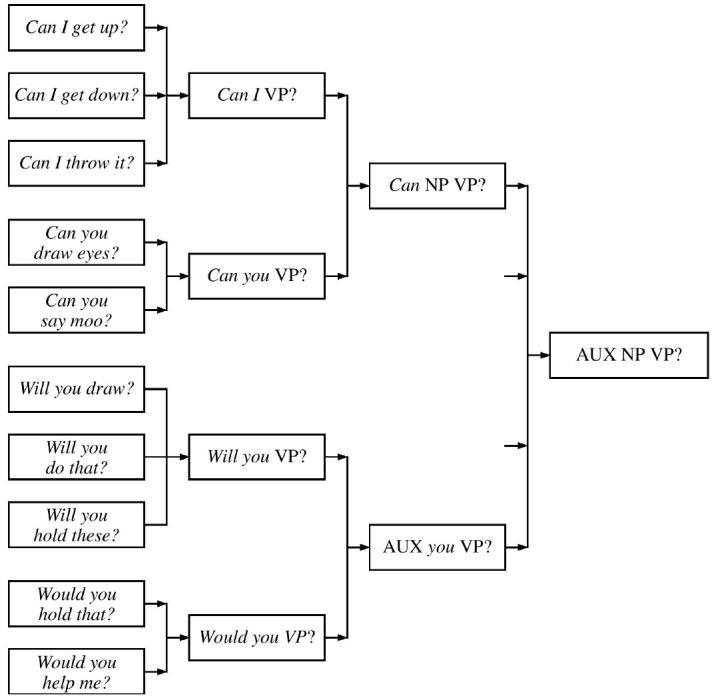
**Progressive schematization.** Labels like NP are VP in the figure are used merely for convenience: we need not assume that the child has abstract syntactic categories, particularly in the early stages of acquisition. The slots in early formulas are defined in semantic terms and may be frame specific, e.g., the VP slot in the *Can I VP?* formula can be filled with any expression referring to “something I would like to do.” For ease of exposition, I am also ignoring the difference between grounded (tensed) and untensed verbs.

Thus, Naomi started with some useful formulas such as request for permission (*Can I ACTION?*), request that the addressee do something for her (*Will you ACTION?*), and offers of an object (*Do you want THING?*). These were gradually integrated into a network of increasingly general constructional schemas. The process is depicted schematically in Figure 1. The left hand side of the figure shows the starting point of development: formulaic phrases. The boxes in the second columns represent low-level schemas which result from generalizations over specific formulaic phrases. The schemas contain a slot for specifying the type of activity; this must be filled by a verb phrase containing a plain verb. The schemas in the third column are even more abstract, in that they contain two slots, one for the activity and one for the agent; they can be derived by generalizing over the low-level schemas. Finally, on the far right, we have a fully abstract Y/N question schema. The left-to-right organization of the figure represents the passage of time, in the sense that concrete schemas developmentally precede more abstract ones. However, the columns are not meant to represent distinct stages, since the generalizations are local: for example, Noami acquired the *Can NP VP?* schema about 6 months before she started to produce *Will you VP?* questions. Thus, different auxiliaries followed different developmental patterns, and, crucially, there is no evidence that she derived questions from structures with declarative-like word order at any stage, as auxiliaries in declaratives were used in very different ways. It is also important to note that the later, more abstract schemas probably do not replace the early lexically specific ones: there is evidence that the two continue to exist side by side in adult speakers (Langacker, 2000; Dąbrowska, 2010b).

Dąbrowska and Lieven (2005), using data from eight high-density developmental corpora, show that young children’s novel questions can be explained by appealing to lexically specific units which can be derived from the child’s linguistic experience. Dąbrowska (2014) argues that such units can also account for the vast majority of adult utterances, at least in informal conversation.

One might object that, since the slots in the formulas can be filled by words or phrases, this approach assumes that the child knows something about constituency. This is true; note, however, that constituency is understood differently in this framework: not as a characteristic of binary branching syntactic trees with labeled nodes, but merely an understanding that some combinations of words function as a unit when they fill a particular slot in a formula. In the constructionist approach, constituency is an emergent property of grammar rather than something that is present from the start, and it is sometimes fluid and variable (cf. Langacker, 1997). Constituency in this sense—i.e., hierarchical organization—is something that is a general property of many cognitive structures and is not unique to language.

### Understanding Language, Warts, and All

Languages are shot through with patterns. The patterns exist at all levels: some are very general, others quite low-level. Languages are also shot through with idiosyncrasies: constructional idioms, lexical items which do not fit easily into any grammatical class, irregular morphology. The generative program focuses on uncovering the deepest, most fundamental generalizations, and relegates the low-level patterns and idiosyncrasies—which are regarded as less interesting—to the periphery. But low-level patterns are a part of language, and a satisfactory theory of language must account for them as well as more general constructions.

Construction grammar began as an attempt to account for constructional idioms such as the *X-er the Y-er* (e.g., *The more the merrier*; *The bigger they come, the harder they fall*—see Fillmore et al., 1988) and *what’s X doing Y?* (e.g., *What’s this fly doing in my soup?*, *What are you doing reading my diary?*—see Kay and Fillmore, 1999). Such constructional idioms have idiosyncratic properties which are not predictable from general rules or principles, but they are productive: we can create novel utterances based on the schema. As construction grammar developed, it quickly became apparent that whatever mechanisms were required to explain low-level patterns could also account for high-level patterns as a special case: consequently, as Croft (2001) put it, “the constructional tail has come to wag the syntactic dog” (p. 17). As suggested earlier, the same is true of acquisition: the learning mechanisms that are necessary to learn relational words can also account for the acquisition of more abstract constructions.

### Back to Poverty of the Stimulus

It is important to note that the way the poverty-of-the-stimulus problem is posed (e.g., “how does the child know that the auxiliary inside the subject cannot be moved?”) presupposes a generative account of the phenomena (i.e., interrogatives are derived from declarative-like structures by moving the auxiliary). The problem does not arise in constructionist accounts, which do not assume movement.

More generally, generativist and constructionist researchers agree about the basic thrust of the POS argument: the child cannot learn about the properties of empty categories, constraints on extraction, etc., from the input. What they disagree about is the conclusion that is to be drawn from this fact. For generative researchers, the fact that some grammatical principles or notions are unlearnable entails that they must be part of an innate UG. Constructionist researchers, on the other hand, draw a completely different conclusion: if X cannot be learned from the input, then we need a better linguistic theory—one that does not assume such an implausible construct.

Thus, one of the basic principles of the constructionist approach is that linguists should focus on developing “child-friendly” grammars (Langacker, 1987, 1991, 2008; Goldberg, 2003; Tomasello, 2003, 2006; Dąbrowska, 2004) rather than postulate an innate UG. Construction grammar attempts to capture all that speakers know about their language in terms of constructions—form-meaning pairings which can be simple or complex and concrete or partially or entirely schematic (i.e., they can contain one or more “slots” which can be elaborated by more specific units, allowing for the creation of novel expressions). Most construction grammar researchers also assume that children prefer relatively concrete, lexically-specific patterns which can be easily inferred from the input; more schematic patterns emerge later in development, as a result of generalization over the concrete units acquired earlier (Johnson, 1983; Dąbrowska, 2000b; Tomasello, 2003, 2006; Diessel, 2004). Crucially, the mechanisms required to learn constructional schemas are also necessary to acquire relational terms such as verbs and prepositions (Dąbrowska, 2004, 2009). Since we know that children are able to learn the meanings and selectional restrictions of verbs and prepositions, it follows that they are able to learn constructional schemas as well.

## Conclusion

As we have seen, contemporary views on what is or is not in UG are wildly divergent. I have also argued that, although many arguments have been put forward in favor of some kind of an innate UG, there is actually very little evidence for its existence: the arguments for the innateness of specific linguistic categories or principles are either irrelevant (in that they are arguments for general innateness rather than linguistic innateness), based on false premises, or circular.

Some generative linguists respond to criticisms of this kind by claiming that UG is an *approach* to doing linguistics rather than a specific hypothesis. For example, Nevins et al. (2009) in their critique of Everett’s work on Pirahã, assert that

“The term Universal Grammar (UG), in its modern usage, was introduced as a name for the collection of factors that underlie the uniquely human capacity for language—whatever they may turn out to be …. There are many different proposals about the overall nature of UG, and continuing debate about its role in the explanation of virtually every linguistic phenomenon. Consequently, there is no general universal-grammar model for which [Everett’s claims] could have consequences – only a wealth of diverse hypotheses about UG and its content.” (p. 357)

This view contrasts sharply with other assessments of the UG enterprise. Chomsky (2000a), for instance, claims that the Principles and Parameters framework was “highly successful” (p. 8), that it “led to an explosion of inquiry into a very broad range of typologically diverse languages, at a level of depth not previously envisioned” (Chomsky, 2004, p. 11), and that it was “the only real revolutionary departure in linguistics maybe in the last several thousand years, much more so than the original work in generative grammar” (Chomsky, 2004, p. 148). If Nevins et al. (2009) are right in their assertion that the UG literature is no more than a collection of proposals which, as a set, do not make any specific empirical predictions about languages, then such triumphalist claims are completely unjustified.

Is it a fruitful approach? (Or perhaps a better question might be: Was it a fruitful approach?) It was certainly fruitful in the sense that it generated a great deal of debate. Unfortunately, it does not seem to have got us any closer to answers to the fundamental questions that it raised. One could regard the existing disagreements about UG as a sign of health. After all, debate is the stuff of scientific inquiry: initial hypotheses are often erroneous; it is by reformulating and refining them that we gradually get closer to the truth. However, the kind of development we see in UG theory is very different from what we see in the natural sciences. In the latter, the successive theories are gradual approximations to the truth. Consider an example discussed by Asimov (1989). People once believed that the earth is flat. Then, ancient Greek astronomers established that it was spherical. In the seventeenth century, Newton argued that it was an oblate spheroid (i.e., slightly squashed at the poles). In the twentieth century, scientists discovered that it is not a perfect oblate spheroid: the equatorial bulge is slightly bigger in the southern hemisphere. Note that although the earlier theories were false, they clearly approximated the truth: the correction in going from “sphere” to “oblate spheroid,” or from “oblate spheroid” to “slightly irregular oblate spheroid” is much smaller than when going from “flat” to “spherical.” And while “slightly irregular oblate spheroid” may not be entirely accurate, we are extremely unlikely to discover tomorrow that the earth is conical or cube-shaped. We do not see this sort of approximation in work in the UG approach: what we see instead is wildly different ideas being constantly proposed and abandoned. After more than half a century of intensive research we are no nearer to understanding what UG is than we were when Chomsky first used the term.

This lack of progress, I suggest, is a consequence of the way that the basic questions are conceptualized in the UG approach, and the strategy that it adopts in attempting to answer them. Let us consider a recent example. Berwick et al. (2011) list four factors determining the outcome of language acquisition:

(1) innate, domain-specific factors;
(2) innate, domain-general factors;
(3) external stimuli;
(4) natural law.

They go on to assert that the goal of linguistic theory is to explain how these factors “conspire to yield human language” (p. 1223), and that “on any view, (1) is crucial, at least in the initial mapping of external data to linguistic experience” (p. 1209).

There are three problems with this approach. First, it *assumes* that innate language-specific factors are “crucial.” It may well be that this is true; however, such a statement should be the outcome of a research program, not the initial assumption.

Secondly, Berwick et al. (2011) appear to assume that the four types of factors are separate and isolable: a particular principle can be attributed to factor 1, 2, 3, or 4. The problem is that one cannot attribute specific properties of complex systems to individual factors, since they emerge from the interaction of various factors (Elman et al., 1996; Bates, 2003; MacWhinney, 2005). Asking whether a particular principle is “innate” or due to “external stimuli” is meaningless—it is both: genes and the environment interact in myriad ways at different levels (molecular, cellular, at the level of the organism, and in the external environment, both physical and social). Asking whether something is “domain general” or “domain specific” may be equally unhelpful. Presumably everybody, including the staunchest nativists, agrees that (the different components of) what we call the language faculty arose out of some non-linguistic precursors. Bates (2003) argues that language is “a new machine built out of old parts”; she also suggests that the “old parts” (memory consolidation, motor planning, attention) “have kept their day jobs” (Bates, 1999). However, it is perfectly possible that they have undergone further selection as a result of the role they play in language, so that language is now their “day job,” although they continue to “moonlight” doing other jobs.

Finally, Berwick et al. (2011) like most researchers working in the UG tradition, assume that one can determine which aspects of language can be attributed to which factor by ratiocination rather than empirical enquiry: “the best overall strategy for identifying the relative contributions of (1–4) to human linguistic knowledge is to formulate POS arguments that reveal a priori assumptions that theorists can reduce to more basic linguistic principles” (p. 1210). This “logical” approach to language learnability is a philosophical rather than a scientific stance, somewhat reminiscent of Zeno’s argument that motion could not exist. Zeno of Elea was an ancient Greek philosopher who “proved,” through a series of paradoxes (Achilles and the tortoise, the dichotomy argument, the arrow in flight), that motion is an illusion. However, Zeno’s paradoxes, intriguing as they are, are not a contribution to the study of physics: in fact, we would not have modern physics if we simply accepted his argument.

Virtually everyone agrees that there is something unique about humans that makes language acquisition possible. There is a growing consensus, even in the generativist camp, that the “big mean UG” of the Principles and Parameters model is not tenable: UG, if it exists, is fairly minimal,^5^ and most of the interesting properties of human languages arise through the interaction of innate capacities and predispositions and environmental factors. This view has long been part of the constructivist outlook (Piaget, 1954; Bates and MacWhinney, 1979; Karmiloff-Smith, 1992; MacWhinney, 1999, 2005; O’Grady, 2008, 2010), and it is encouraging to see the two traditions in cognitive science are converging, to some extent at least.

The great challenge is to understand exactly how genes and environment interact during individual development, and how languages evolve and change as a result of interactions between individuals. To do this, it is crucial to examine interactions at different levels. Genes do not interact with the primary linguistic data: they build proteins which build brains which learn to “represent” language and the external environment by interacting with it via the body. It is unlikely that we will be able to tease apart the contribution of the different factors by ratiocination: the interactions are just too complex, and they often lead to unexpected results (Thelen and Smith, 1994; Elman et al., 1996; Bates, 2003; MacWhinney, 2005). We have already made some headway in this area. Further progress will require empirical research and the coordinated efforts of many disciplines, from molecular biology to psychology and linguistics.

### Conflict of Interest Statement

The author declares that the research was conducted in the absence of any commercial or financial relationships that could be construed as a potential conflict of interest.

## Footnotes

1 Our nearest relatives, the great apes, do not point and do not understand pointing gestures (Tomasello, 1999; Tomasello et al., 2005). Dogs do understand human pointing, which is believed to be a consequence of domestication (Hare et al., 2002); they do not, however, use pointing gestures themselves. “Pointing” dogs do not intentionally point things out to others: they merely look at the game, enabling the human hunter to follow their line of sight.

2 The Manchester corpus is described in Theakston et al. (2001) and is available from CHILDES (MacWhinney, 1995).

3 It should be emphasized that these styles are idealizations. Most children use a mixture of both strategies, although many have a clear preference for one or the other.

4 Naomi’s linguistic development was recorded by Sachs (1983). The transcripts are available from the CHILDES database (MacWhinney, 1995).

5 In fact, Roberts and Holmberg (2011) suggest that “UG does not have to be seen as either language-specific or human-specific,” thus capitulating on the central claims of the UG approach. Note that this dilutes the innateness hypothesis to the point where it becomes trivial: if UG is neither language specific nor human specific, then saying that it exists amounts to saying that we are different from rocks.
